# Cross-species conversations: the development of the AURAL-Pet coding system to capture how people talk to pets in daily life

**DOI:** 10.3389/fpsyt.2026.1881870

**Published:** 2026-08-04

**Authors:** Dara S. Jonkoski, Nicole M. Lorig, Molly C. Delzio, Alyssa Klensin, Abigail Marsters, Huashi Li, Amanda M. Bernal, Matthias R. Mehl, Kerri E. Rodriguez, Emily E. Bray

**Affiliations:** 1 University of Arizona, College of Veterinary Medicine, Tucson, AZ, United States; 2 University of Arizona, Department of Psychology, College of Science, Tucson, AZ, United States; 3 University of Arizona, Cognitive Science Program, College of Science, Tucson, AZ, United States; 4 University of Arizona, Center for Biomedical Informatics and Biostatistics, Statistics Consulting Laboratory, Tucson, AZ, United States

**Keywords:** coding system, ecological momentary assessment, ethogram, human-animal interaction, inter-rater reliability, pet-directed speech

## Abstract

While pets are a well-established fixture in many households, how owners perceive their pets’ roles can range from distant housemates to casual companions to trusted confidantes. At the same time, findings relating to the impact pets have on human health and wellbeing are mixed. A potential explanation for these disparate effects is the naturally occurring variation in daily human-pet interactions and relationship quality. However, there is a lack of methodological approaches that can accurately quantify naturalistic interactions between people and their pets with limited bias. To address this gap, we developed the Auditory-based Understanding of Relational Animal-directed Language (AURAL-Pet) coding system to apply to ambient sound clips of human-pet interactions as captured by the Electronically Activated Recorder (EAR). By consulting existing literature, subject matter experts, and an archival database of EAR audio clips, we used an iterative process to develop and refine the coding system. In the final version, we retained 30 variables, 27 binary and 3 qualitative, which extract aspects of pet-focused activities, as well as the quality and content of human speech directed towards pets. We found that 22 out of 23 original AURAL-Pet binary variables had acceptable inter-coder agreement (ICC ≥.70). We then applied AURAL-Pet to four archival EAR studies, totaling N = 5,072 ambient audio clips containing evidence of pet presence across N = 240 participants, to better understand how frequently participants demonstrated different human-pet interactions across gender- and age-diverse samples. We found that individual participants’ interactions with pets varied extensively across all variables, and that certain variables were more heterogeneous across genders and ages than others. In summary, this coding tool provides a novel, reliable method for quantifying daily human-pet interactions across diverse populations. Future work applying AURAL-Pet to naturalistic audio clips would be well-positioned to explore how differences in human-animal interactions may impact both human and animal health and wellbeing.

## 1 Introduction

Pets have been shown to play an important role in many people’s daily lives. A survey analyzing pet ownership globally across more than 20 countries found that on average, one third of households own a dog, and almost one fourth own a cat (1). Meaningful bonds shared between human-pet dyads are commonplace and widely acknowledged. For example, a majority of pet owners in the United States view their pet as a family member (2, 3). Accordingly, a growing body of research has explored the relationship between pet ownership and human health and wellbeing (4). However, findings related to the “pet effect” have been mixed (5, 6). For example, some studies find that pet ownership is associated with positive health outcomes, including less loneliness and depression (7, 8). Yet, other studies find that pet ownership has little to no effect on human health and wellbeing outcomes (9–11).

It is possible that individual differences in pet-owner interactions may play a role in these discrepant findings by moderating the benefits associated with pet ownership (12). Recent research has shifted away from exploring the relationship between pet ownership and human wellbeing as a binary variable (i.e., comparing those who have pets to those who do not), instead opting to look within pet owners to ask how the *quality* and *quantity* of daily pet interactions impact wellbeing on a more granular level (4, 13, 14). Studying the nuanced ways in which individuals interact with their pets can help uncover the specific contexts in which pet ownership may, or may not, support human wellbeing.

One method that researchers have used to better understand daily pet interactions is ecological momentary assessment (EMA) techniques (15–21). EMA involves repeated sampling of participants’ current behaviors and experiences in their natural environments (22). Typical EMA studies—including those investigating human-pet interactions—often use short surveys administered through mobile phone apps or smart watches to capture people’s in-the-moment thoughts and behaviors. This approach not only minimizes recall bias but also provides representative assessments of everyday life (23). EMA methods can also allow researchers to measure how being in the presence of social companions, including pets, is related to momentary mood and wellbeing (21). However, EMA methods still face challenges when considering validity and generalizability, as even in-the-moment self-reports can still be subject to self-report biases. For example, responses can be biased due to a lack of attention and memory (e.g., not remembering what exactly was said) or the self’s vantage point in the assessment (e.g., self-deception and socially desirable responding; 24).

To overcome self-report biases, researchers can use observational methods to quantify pet owners’ daily interactions with their pets. One pet-directed behavior receiving increased research interest is the intonation people adopt when talking to their pets. Studies on the pet-directed speech register—a vocal register similar to infant-directed speech—have found that various factors shape how people speak to their pets. These factors include the speaker’s gender (25–28), personality (25), parental status and perceived level of social support (29), and the context of the interaction (25, 30). However, the literature in this area has yet to agree on a single definition of the pet-directed speech register. Some studies define it in terms of acoustic content (27, 30), while others focus on pitch and/or affect (25, 26, 29). This inconsistency demonstrates the need for a standardized approach to measuring the pet-directed speech register. This standardization is especially important given that the pet-directed speech register is nuanced and shaped by the situational context of the actors and the interaction itself.

In addition, measuring the different ways in which individuals talk to their pets may reveal unique insights into the nature of daily pet-owner interactions, as well as the impact of those interactions on both the people and pets involved. And yet, researchers still have a limited understanding of the variation in how people talk to their pets, including not just in register but also in content, and the factors that contribute to those differences. One potential limitation lies in the methodologies and designs used. Many studies that analyze speech directed towards pets have focused on specific interactions, such as playing with or walking the animal (25–27, 30–34). Other studies have recorded pet owners in unfamiliar or unnatural environments, such as a laboratory or veterinary space (25, 27, 30, 34, 35). These contexts, while important and informative, do not represent the wide array of daily interactions that occur in human-pet dyads. Studies which can measure behavior in familiar environments over prolonged periods are best suited to capturing daily pet interactions. Thus, in the current study we aimed to build on past literature by listening to human-pet interactions via a minimally intrusive and ecologically valid method in just those sorts of contexts.

The electronically activated recorder (EAR) is an observational EMA method that could be uniquely advantageous in understanding the nuances of daily pet interactions on a larger and more naturalistic scale than has been previously attempted. The EAR is a smartphone application that intermittently (e.g., randomly five times per hour) and passively (i.e., without the participant being aware of the exact times) records ambient sound throughout the day over multiple days (36). The EAR has been deployed as a psychometrically valuable momentary measurement tool in social and personality psychology studies since 2001 (37). Because participants are unaware of when recordings take place, the EAR engages self-presentation mechanisms (e.g., self-censoring via behavioral inhibition or accentuation) less than methods that require participants to activate their own recordings (29) or that rely on direct observation by physically present researchers or video recordings (33, 38). As evidence of this, the EAR has been successfully used to study subtle and habitual behaviors, such as swearing (39) and sighing (40, 41), and can quantify behaviors that participants either may not pay attention to, consider irrelevant to report, or remember in a selective way (36). Furthermore, participants’ self-awareness in wearing the EAR device fades within the first 2 hours of wearing it (42). Thus, the EAR represents a days-long, naturalistic observational method that is novel to human-animal interaction studies. When interpreted through the lens of a pet-specific coding system, it can capture the intricacies of daily speech and interactions between people and their pets, including many that would likely not emerge in a contrived environment. This is particularly relevant as researchers seek to clarify for whom and in which contexts pet ownership may be beneficial for both human and animal wellbeing (43)—a question that the EAR is uniquely suited to help address.

While the EAR has been extensively used to quantify and describe people’s daily social behavior across a variety of contexts and populations (e.g., 44–48), it has never previously been used to quantify people’s social behavior with pets. The primary goal of the current study was to develop and establish the reliability of a coding system to describe daily human-pet interactions and speech (specifically, with cats and dogs) captured by EAR recordings, which we refer to as AURAL-Pet (Audio-based Understanding of Relational Animal-directed Language). As a secondary, exploratory goal, we applied AURAL-Pet to a set of archival EAR studies to provide initial descriptive statistics characterizing the frequency and variability of pet-directed interactions.

## 2 Materials and equipment

The AURAL-Pet coding system was developed using archival data derived from four EAR studies using different versions of the EAR device (36). Information on EAR-specific hardware and software can be found at Megan Robbins’s Open Science Foundation (OSF) Ear Repository (https://osf.io/n2ufd/). However, it is our intention that the coding system can be applied to any auditory interactions that humans have with dogs or cats, so in practice any device which can capture audio data may be used.

## 3 Methods

**Figure 1:**
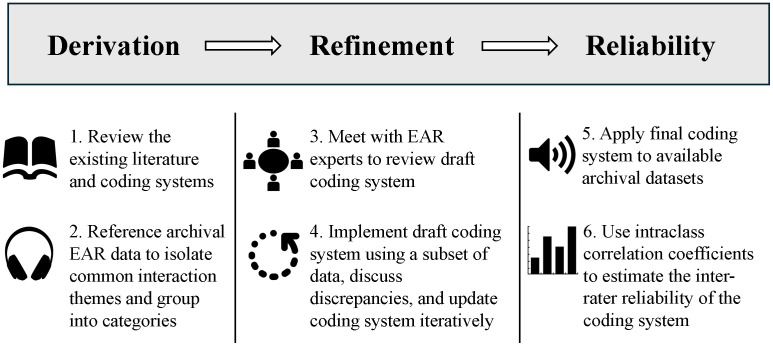
High-level overview of the AURAL-Pet creation process. Icons were obtained from Wikimedia Commons and are available under the CC0 1.0 Universal Public Domain Dedication: “Open Book” by Maki contributors; “Headphones” by Yohann Berger; “Meeting” by Melissa Gaviria; “Update” and “Audio Max” by the Consumer Financial Protection Bureau; and “Bar Graph” by Ben King.

To develop AURAL-Pet, we used archival data from four previously published projects using the EAR among various adult populations based in the United States (older adults [Study A – Aging]: 49; healthy adults practicing meditation [Study B – CALM]: 50, 51; couples coping with breast cancer [Study C – Cancer]: 52–54; and recently divorced and separated adults [Study D – DSE]: 55–58). The included studies were approved by the University of Arizona Institutional Review Board (IRB# 1803376749). These studies were conducted in accordance with the local legislation and institutional requirements. Guided by the general protocol outlined in Kaplan et al. (59), we used a systematic approach to derive, iteratively refine, and then test the reliability of AURAL-Pet (**Figure 1**).

### 3.1 Step 1. Top-down process: review the existing literature and coding systems

Our first step was to review previous studies which quantified spoken interaction with pets, as well as studies investigating the impact of pets on the wellbeing of humans with whom they interact (including specific hypothesized mechanisms), to start determining which behavioral variables would be conceptually interesting to include based on the available literature and theory. Three researchers used Google Scholar to search keywords relating to speech directed towards pets (e.g., “talking to pets”, “pet-directed speech”, etc.). We identified studies describing the ways in which humans typically talk to animals (e.g., 26, 27, 30–34, 38, 60), and studies describing the type of verbal communication that the pets themselves were most likely to attend to (e.g., 61–64). From these studies, we took high-level notes about the specific behaviors and speech types the authors captured from their samples. To locate studies that detailed the potential impacts of pet interaction and ownership (e.g., 65–74), we once again searched Google Scholar using keywords relating to human-pet interaction and mechanisms behind the benefits of pet ownership (e.g., “human-animal bond”, etc.) and further explored those studies’ citations and references to get a deeper understanding. Through exploring these mechanisms, we wanted to ensure that we included variables in AURAL-Pet that could ultimately address evidence-based hypotheses for the underlying mechanisms that may explain the impact of pet ownership. For the current coding system, we drew on several theoretical perspectives to guide variable development, including biophilia, social support theory, and the biopsychosocial model (4, 12). All of these studies helped our team conceptualize variables in the early stages of AURAL-Pet.

Some previous studies were conducted via in-person observations or via video recordings, which allowed researchers to measure verbal and non-verbal communication (27, 34, 38, 75). Additionally, many studies were conducted in experimental settings, such as during a playful interaction, or during a walk (26, 27, 30–34, 38, 60), meaning these coding systems did not include the full of spectrum variables which could capture a wide range of naturalistic interactions that occur throughout an entire day. Additionally, many studies recorded human-pet interactions for multiple consecutive minutes, in contrast to the short, intermittent audio clips that the EAR captures. Because of all these contextual differences, we also needed to base some variables on specific speech and activity patterns we heard in archival EAR data.

### 3.2 Step 2. Bottom-up process: reference archival EAR data to isolate common interaction themes and group into categories

To complement the theory-driven approach, we also took a data-driven, bottom-up approach to isolate common themes from the archival EAR data. To do so, a core member of the research team listened to a random subset of the archival EAR data to identify aspects of the clips that could feasibly be coded and that represented the variability in quality (i.e., positive or negative valence), depth (e.g., the length and level of detail of speech directed towards pets), and type (i.e., speech or activity patterns) of human-pet interactions. We specifically wanted to capture behaviors and types of speech which were observed frequently both within and between participants.

### 3.3 Step 3. Meet with EAR experts to review draft coding system

After drafting an initial version of AURAL-Pet [See: **Supplementary Table 1** (Original AURAL-Pet Coding System)], two EAR coding system experts were enlisted to provide feedback on the feasibility of the initial variables. The primary feedback was that the coding system was too large (at 55 variables), which could lead to coder fatigue and thereby reduce reliability. The two EAR experts and the primary coding system creator then implemented an iterative process to refine the coding system over several rounds. For example, we originally differentiated between clips in which the owners were positive, annoyed, apologetic, sarcastic, and neutral. However, the EAR coding system experts advised that specific affective tones are notoriously difficult to code from auditory data reliably (76, 77), especially when further complicated by sarcasm and irony (i.e., a discrepancy between verbal sentiment and non-verbal tone). Because we were in the process of cutting down the number of variables in the coding system regardless, we instead settled on a simpler path by quantifying whether the contents of the words spoken to the pets implied that the person felt the pet was being “good” (i.e., “positive appraisal”) or “bad” (i.e., “negative appraisal”). As we still wanted to capture the valence of interactions, we anticipated that this distinction was a compromise which maintained practicality for coding efficiently and the ability to capture differences in human-pet interaction styles between participants (i.e., by quantifying whether participants had more “positive” or “negative” interactions with their pets). During the revision process, we prioritized creating a coding system that would be the most intuitive, carefully balancing the desire for the coders to spend minimal time making categorization determinations while still ensuring that important information regarding the quality of the pet interactions was sufficiently captured. By the end of this revision process, the unfinalized second version of the coding system consisted of 30 variables [see: **Supplementary Table 2** (First Applied Version of AURAL-Pet)].

### 3.4 Step 4. Revision: implement draft coding system using a subset of data, discuss discrepancies, and update coding system iteratively

Next, 169 audio clips (Subset 1) from nine participants across the four archival data sets (49, 50, 52, 55) were selected non-randomly to capture variability in all variables. The primary coding system creator cultivated this dataset by listening to clips from participants across all four datasets until they found a representative sample of multiple examples of each variable, which would allow independent coders to test the feasibility of using the full coding system on real data. Five coders were trained on an early version of AURAL-Pet, which consisted of attending an interactive presentation in which they learned the definitions of each of the coding system’s variables and practiced applying them to 12 audio clips together. The five coders then independently applied this version of AURAL-Pet to Subset 1. Using feedback from these coders iteratively as they worked through the data, the coding system was refined by the authors through enhancing definitions of existing variables to either clarify details or loosen criteria (i.e., creating less strict criteria for variables to decrease subjectivity in the variable definitions and increase agreement between coders), adding new variables to capture content that was missing from the original system (e.g., the variable, “question”, which was suggested by an author to further capture the complexity of speech towards pets, and the variables “pitch change”, “slower tempo” and “vowel modulation”, which were derived from our original “pet-directed speech register” variable after we found it was too difficult to capture reliability when each aspect was required to be included under one variable), and removing variables found to be too difficult to establish from an audio clip lasting less than one minute. Generally, it was our goal to refine, create, and remove variables so that our coding system could be reliably applied by independent coders, while still capturing a variety of behaviors that could potentially elucidate how different interaction styles map onto different pet owner mental health and wellbeing outcomes.

Once we felt the revised coding system was in a place where it could be initially tested for inter-rater reliability, a new subset of 188 novel clips (Subset 2) from 14 participants across the four archival data sets were non-randomly selected to again capture all AURAL-Pet variables (no clips from Subset 1 were repeated) by the primary coding system creator using the same process they used to select Subset 1. We used two-way random intraclass correlations (ICC [2,2]) to estimate the reliability between two coders (who marked each binary variable as either “present” or “absent”) on Subset 2. Variables which did not meet the threshold for acceptable reliability (ICC ≥.70, as is standard for variables quantifying EAR data; 42, 59) were reviewed and discussed. The coding system creators updated the definitions for these variables and had the coders re-code all the data using the updated definitions, and repeated the above process [i.e., updating variables and calculating ICC (2,2) until reliability on all variables exceeded the .70 threshold].

### 3.5 Step 5. Apply final coding system to available archival datasets

As the culminating step in our process, the same two coders applied the final refined coding system including 30 variables (27 binary and 3 qualitative) to every pet-adjacent clip (i.e., those determined by the archival coders to have a pet present either from pet sounds of life or someone referencing the pet directly) from the four archival datasets. We excluded any clips in which at least one coder believed a language other than English was spoken for a majority of the time (n = 3 clips), as well as the pet-tagged clips for a participant with only a bird present (n = 55 clips), since AURAL-Pet was designed to capture interactions with cats and dogs only. Once both coders finished coding all data from one study (Study A - Aging; 49) in its entirety, reliability was once again estimated using ICC (2,2). The results indicated that all but six variables were over threshold (ICC ≥.70). After discussion, we further refined definitions of the underperforming variables to minimize subjective bias. For example, definitions for the variables “positive appraisal” and “negative appraisal” originally contained assessment of both content and tone, but our updated definition instructed coders to base their coding decision on content alone, regardless of tone of voice (as even distinguishing between positive and negative tone proved too difficult, see: “Step 3”).

With these updated definitions, clips across all four archival datasets marked by either coder as including any of these six variables were revisited to ensure proper coding. Due to time constraints, one coder was unable to recode these variables, and so a third coder was recruited and trained to do so. The resulting dataset was fully coded with the final version of AURAL-Pet (**Table 1**).

**Table 1:** Finalized AURAL-Pet coding system.

Level	Variable	Variable definition	Notes
Social context	Alone	Participant is by themselves. Also applies if participant is surrounded by people, but not engaged in any interaction (e.g., by themselves in a coffee shop)	Mutually exclusive with any of the ‘With others’ options
	With others (one)	Participant is with ONE other person, and they are engaged in a conversation, interaction, or joint activity of some kind (e.g., watching a movie, gaming)	Mutually exclusive with ‘Alone’ and ‘With others (multiple)’
	With others (multiple)	Participant is with MORE THAN ONE other person, and they are engaged in a conversation, interaction, or joint activity of some kind (e.g., watching a movie, gaming)	Mutually exclusive with ‘Alone’ and ‘With others (one)’
	With pet	Pet (either a dog or cat) is present (irrespective of whether the pet is right next to or nearby the participant); includes scenarios in which multiple pets are present. Indicated by either pet sounds of life, or someone referencing the pet directly (e.g., stating the pet’s name, a variation of the pet’s name, a common nickname like “baby” or “my love”, and/or using language typically directed towards a pet when it is not obviously directed at another human)	
Activity	Pet-focused activity (PFA)	Participant is engaged in an activity focused on a pet	If yes, one of the three below options must also be yes
	PFA - Playing	Participant is playing with pet; must be physically involved in the activity to count (e.g., playing tug of war, dangling toy in front of cat, play wrestling with dog, playing fetch, playing chase)	Mutually exclusive with ‘PFA - Going on walk’ and ‘PFA - Other activity’
	PFA - Going on walk	Participant and pet are accompanying each other on a walk outdoors	Mutually exclusive with ‘PFA - Playing’ and ‘PFA - Other activity’
	PFA - Other activity	Participant engaged in an activity (other than those listed above) focused on a pet	Mutually exclusive with ‘PFA - Going on walk’ and ‘PFA - Playing’; Exact ‘other activity’ recorded as a free response
General vocalizations	Pet vocalization	Pet vocalization present (e.g., “whine”, “bark”, “meow”)	‘Vocalization type’ recorded as a free response
	Talking	Participant is talking (in person, on the phone, via video chat or live chat). Includes non-fluencies. This includes talking to oneself and talking to a pet	
	Talking about pet	Participant speaks to another human about a pet. Includes instances in which the participant doesn’t actually say anything about the pet, but is actively involved in a conversation about the pet (i.e., responding to something said about the pet)	
Speech directed towards pets	Talking to pet	Participant speaks directly to pet (can be word or other sound); includes scenarios in which the participant is talking to multiple pets	If ‘yes’, ‘Animal content’ and/or ‘Human substitute’ must be ‘yes’
	Animal content	Participant talks to the pet as an animal [includes greetings, (e.g., “how are you”), any speech for reasons instrumental to the health and wellbeing of the pet (e.g., food-related, waste elimination-related), unless the instrumental-related speech goes past basic needs (e.g., asking a pet which brand of food they’d prefer), low-level descriptions about the pet’s appearance/personality/behaviors, which are non-metaphorical (e.g., includes words like “pretty”, “silly”, “cute”, “good”; excludes words like “priceless” and “killer”), non-word sounds]	
	Human substitute (HS)	Participant converses with the pet as if it was a human (e.g., expressing complex ideas that a pet could not comprehend, speech going past health-related instrumental purposes)	‘…About What?’ recorded as a free response with keywords or short phrases; If yes, either ‘Brief remark’ or ‘Extended remark’ must also be yes
	HS - Brief remark	Short remark only; up to two phrases or sentence fragments; phrases that are repeated EXACTLY are treated as a single phrase (e.g., “You are my angel. You are my angel.” counts as one phrase; “My silly angel dog. You are my angel.” counts as two phrases because repetition is not exact)	Mutually exclusive with ‘HS - Extended remark’
	HS - Extended remark	Anything longer than two phrases or sentence fragments; phrases that are repeated EXACTLY are treated as a single phrase	Mutually exclusive with ‘HS - Brief remark’
	Command	Participant uses a sentence that gives a command (e.g., “get the ball”, “go away”, etc.); command aims at controlling the pet’s behavior, can be standard command or idiosyncratic variation in pitch or wording, including phrased as a question (e.g., “Do you want to get me that ball?”)	Inclusion of idiosyncratic variation in pitch (inspired by “imperatives disguised as questions”; Mitchell & Edmonson (32))
	Question	Participant uses a sentence that aims to elicit information from the pet (e.g., “who’s a good girl”, “who knocked this over?”, “who wants to go for a walk?”), even if the pet isn’t able to literally provide an answer; includes statements that end in question tags (e.g., “you’re enjoying that toy, aren’t you?”)	
	Positive appraisal	Participant verbally expresses to the pet that the pet is good/did something right using phrases typically used to express praise (e.g., “good boy”, “I love you”, “you’re so cute”); positive tone alone is not sufficient	
	Negative appraisal	Participant verbally expresses to the pet that the pet is bad/not fine/did something wrong using phrases typically used to express dissatisfaction (e.g., “no”, “don’t do that”, “stop it”, etc.); negative tone alone is not sufficient	
	Pet-directed speech register (PDSR)	Participant uses at least one aspect of the pet-directed speech register (e.g., with high and variable pitch, slower tempo, and/or modulation to the pronunciation of vowels)	If yes, ‘PDSR - Pitch change’, ‘PDSR - Slower tempo’, and/or ‘PDSR - Vowel modulation’ must also be yes
	PDSR - Pitch change	Participant noticeably modulates their pitch; high and variable pitch, regardless of how much pitch is modulated in adult-directed speech	
	PDSR - Slower tempo	Participant speaks at a slow speed	
	PDSR - Vowel modulation	Participant alters the way in which they say their vowels (e.g., hyperarticulation, longer vowel space, elongation of vowel sounds)	
	Irritation/frustration	Participant gives (clear) expression of irritation, frustration, or anger; i.e., “letting it out” on the pet, “losing one’s cool”, unnecessary negativity, or venting grievances going further than corrections for bad behavior	
	Mind reading	Participant vocalizes inferences regarding specific concerns, emotions, needs, wishes, wellbeing, or intentions of the pet; explicitly vocalizing inferences of what the pet might think or feel (must go further than “I know”, e.g., “I know you are hungry” is okay). Can include questions (e.g., “you want to go potty?”)	Inspired by empathic sentences; Rogers et al. (33); and attribution of thought; Sims & Chin (34)
	Pet nickname use	Participant uses an affectionate nickname for pet (e.g., “baby”, “princess”, “big yellow”, some alteration of the pet’s name); does not include “boy” or “girl”, but does include those words when they are preceded by an adjective other than “good”	
	Parental role	Participant refers to themselves or another person as the pet’s parent, or states that the pet is their child or another person’s child (e.g., “mommy’s home”, “come give daddy a kiss”, “mom wouldn’t like that very much”, the owner referring to the pet as the sibling of their human children); does not include the use of the nickname “baby” or “my baby”	

### 3.6 Step 6. Use intraclass correlation coefficients to estimate the inter-rater reliability of the coding system

The final dataset consisted of 5,072 sound clips of either 30- or 50-seconds in length from 240 participants whom the coders from the archival studies determined to have at least 2 pet-adjacent clips. As mentioned previously, a third coder was recruited to ensure the updated six variables were coded according to the updated definition, which was applied consistently across all four archival datasets. Additionally, because four of the variables in AURAL-Pet (“alone”, “with others (one)”, “with others (multiple)”, and “talking”) were previously coded as part of the archival studies and found to be reliable at that time, we utilized the original coding of those variables from the archival datasets rather than recoding them (inter-rater reliability discussed in “Results”). We found that once trained, our coders spent an average of 1.5 minutes applying AURAL-Pet to a single audio clip of either 30- or 50-seconds in length.

To ensure that AURAL-Pet was applied consistently across variables by the coders, we estimated inter-rater reliability using one-way random intraclass correlations for both the average [ICC (1,2)] and single [ICC (1,1)] measure. We estimated the reliability for both the aggregate measure (i.e., the average of the two codings) and a single measure (i.e., the reliability of either one of the two codings individually; 78) to provide future researchers using AURAL-Pet with the psychometric information they need to make informed decisions about which variables might require multiple coders, and which might be appropriate to be single-coded. We calculated one-way ICCs since our coding process (wherein a third coder updated some of another coder’s work) was not fully consistent with a two-way random measurement model (79).

## 4 Results

### 4.1 Inter-rater reliability

Based on the standards set by previous EAR studies regarding acceptable inter-rater reliability (42, 59), we evaluated the reliability of the average measure, that is ICCs (1,2), and found 22 original binary variables had acceptable agreement (ICC ≥.70). When evaluating the reliability of a single coding, that is ICCs (1,1), 14 original binary variables had acceptable agreement (ICC ≥.70; see **Table 2**).

**Table 2:** Inter-rater reliabilities for original binary variables in AURAL-Pet.

Level	Variable	ICC (1,2)	ICC (1,1)
Social context	With pet	.85	.75
Activity	Pet-focused activity (PFA)	.86	.76
	PFA - Playing	.93	.87
	PFA - Going for a walk	.97	.94
General vocalizations	Pet vocalization	.94	.89
	Talking about pet	.90	.82
Speech directed towards pets	Talking to pet	.96	.92
	Animal content	.65	.49
	Human substitute (HS)	.73	.58
	HS - Brief remark	.79	.65
	HS - Extended remark	.79	.65
	Command	.85	.74
	Question	.93	.87
	Positive appraisal	.86	.75
	Negative appraisal	.86	.76
	Pet-directed speech register (PDSR)	.83	.70
	PDSR - Pitch change	.72	.56
	PDSR - Slower tempo	.75	.61
	PDSR - Vowel modulation	.72	.56
	Irritation/frustration	.80	.66
	Mind reading	.76	.61
	Pet nickname use	.87	.76
	Parental role	.92	.84

For variables which were already coded from the archival studies during baseline EAR coding (“talking”, “alone”, “with others (one)”, and “with others (multiple)”), we report the reliability from the original studies (these calculations include non-pet-adjacent data; **Table 3**). Across all variables and studies, agreement exceeded the acceptable threshold (ICC ≥.70).

**Table 3:** Reliability for all variables in AURAL-Pet used from archival data.

Study	Study A - Aging: 49*	Study B - CALM: 50^†^	Study C - Cancer: 52^†^	Study D – DSE: 55^†^
Talking	.98	.98	.97	.99
Alone	.95	.96	.86	.99
With others (one)	.93	.89	.83	.93
With others (multiple)	.97	.90	.90	.74

*Reliability calculated on data subset; ICC (1,2).

^†^ Reliability calculated on entire dataset; ICC (1,2).

### 4.2 Participant demographics

Upon obtaining acceptable reliability and as part of an exploratory aim, we applied AURAL-Pet to four archival studies to characterize the frequency and variability of pet-directed interactions. Across the four datasets, we found that 44% (240/541) of the total participants were pet-adjacent (i.e., there was evidence of pet presence in two or more of their audio files at baseline coding), and thus eligible to contribute data relevant to AURAL-Pet. Of these N = 240 participants, 68.3% (n = 164) were female and 31.7% (n = 76) were male. The participants’ mean age was 48.5 (range: 24 – 87, SD = 17.9; excluding one participant, who did not provide their age). Demographic breakdowns separated by study are also presented in **Supplementary Table 3**.

### 4.3 Frequency of talking to pets among all talking

To account for inter-rater variation, we generated a single score for each variable by averaging across the coders. Furthermore, because each participant varied in the number of sound clips in which our coders identified pet presence (averaged mean: 18.1 clips, range: 0.5 – 123.5 clips, SD = 19.8 clips), we represent the variable frequencies as percentages (note: the minimum number of clips is less than 2 because *n* = 2 participants were determined to have fewer than 2 clips with dog and/or cat presence when assessed by our AURAL-Pet coders).

We quantified how often participants spoke to pets in comparison to the time they spent speaking generally (i.e., including to pets, themselves, another person, or a group of people). To do so, we divided the number of clips in which participants spoke to pets by the number of clips in which participants spoke to anyone (including the N = 5,072 talking clips analyzed using AURAL-Pet as well as all non-pet-adjacent talking clips as well). We found that people spoke to pets in an average of 9.2% (range: 0 – 49.6%, SD = 8.7 percentage points) of the 30,433 clips in which they spoke at all.

### 4.4 Presence of other people

Pets were prevalent in both moments of solitude and within social settings. On average, participants were alone (i.e., not with any other people) in 44.0% (range: 0 – 100%, SD = 32.4 percentage points) of pet-adjacent clips, with one other person in 35.7% (range: 0 – 100%, SD = 27.7 percentage points) of pet-adjacent clips, and with multiple people in 14.1% (range: 0 -100%, SD = 22.3 percentage points) of pet-adjacent clips.

### 4.5 Descriptive statistics for remaining binary variables

The descriptive statistics for each original variable contained in AURAL-Pet are presented in **Table 4**.

**Table 4:** Frequency of each original variable in AURAL-Pet represented as percentages (except for “Vocalization type” and “About what?”, which are descriptors of the content contained in “Pet vocalization” and “Human substitute”, respectively, meaning they have the same frequency as their parent variable; see “Content reported in qualitative variables” for more information about specifics of content reported in all qualitative variables).

Level	Variable	Overall	Female	Male	≤35	36–59	60+
		(N = 240)	(n=164)	(n=76)	(n=76)	(n=100)	(n=63)
Activity	Pet-focused activity (PFA)	9.9 (14.5) (0.0–80.0)	9.8 (15.2) (0.0–80.0)	10.3 (13.1) (0.0–53.1)	9.3 (13.3) (0.0–52.9)	8.7 (13.3) (0.0–53.1)	12.5 (17.6) (0.0–80.0)
	PFA - Playing	0.5 (2.7) (0.0–25.0)	0.5 (2.4) (0.0–25.0)	0.7 (3.3) (0.0–25.0)	0.7 (2.1) (0.0–12.5)	0.5 (2.8) (0.0–25.0)	0.5 (3.2) (0.0–25.0)
	PFA - Going on walk	7.7 (13.7) (0.0–80.0)	7.4 (14.4) (0.0–80.0)	8.2 (12.3) (0.0–50.0)	7.5 (12.2) (0.0–50.0)	7.0 (12.7) (0.0–50.0)	9.1 (16.9) (0.0–80.0)
	PFA - Other activity	1.7 (3.8) (0.0–21.4)	1.9 (4.1) (0.0–21.4)	1.3 (2.8) (0.0–13.6)	1.1 (3.0) (0.0–20.0)	1.3 (3.3) (0.0–19.0)	2.9 (4.7) (0.0–21.4)
General vocalizations	Pet Vocalization	28.1 (24.3) (0.0–100.0)	29.3 (23.7) (0.0–100.0)	25.4 (25.5) (0.0–100.0)	29.5 (23.9) (0.0–100.0)	27.3 (24.7) (0.0–100.0)	27.4 (24.6) (0.0–100.0)
	Talking about pet	19.9 (19.2) (0.0–100.0)	18.3 (18.4) (0.0–100.0)	23.1 (20.7) (0.0–83.3)	25.3 (20.8) (0.0–85.7)	17.0 (16.7) (0.0–66.7)	17.3 (19.8) (0.0–100.0)
Speech directed towards pets	Talking to pet	63.3 (27.9) (0.0–100.0)	64.6 (27.1) (0.0–100.0)	60.4 (29.5) (0.0–100.0)	60.2 (26.1) (0.0–100.0)	66.3 (29.8) (0.0–100.0)	61.9 (26.6) (0.0–100.0)
	Animal content	55.3 (26.0) (0.0–100.0)	56.3 (24.6) (0.0–100.0)	53.1 (28.9) (0.0–100.0)	53.0 (23.3) (0.0–100.0)	58.5 (27.3) (0.0–100.0)	52.3 (26.4) (0.0–100.0)
	Human substitute (HS)	18.2 (18.6) (0.0–100.0)	19.1 (18.2) (0.0–100.0)	16.1 (19.3) (0.0–100.0)	15.0 (15.7) (0.0–75.0)	20.2 (19.0) (0.0–100.0)	19.0 (20.9) (0.0–100.0)
	HS - Brief remark	10.7 (11.4) (0.0–66.7)	11.7 (11.7) (0.0–66.7)	8.7 (10.7) (0.0–50.0)	10.2 (10.6) (0.0–40.0)	12.3 (12.0) (0.0–66.7)	9.0 (11.4) (0.0–50.0)
	HS - Extended remark	7.4 (12.7) (0.0–100.0)	7.4 (10.9) (0.0–50.0)	7.4 (15.9) (0.0–100.0)	4.8 (9.5) (0.0–50.0)	7.9 (11.8) (0.0–50.0)	9.9 (16.4) (0.0–100.0)
	Command	24.7 (20.8) (0.0–100.0)	25.8 (20.8) (0.0–100.0)	22.4 (20.9) (0.0–83.3)	25.7 (20.3) (0.0–100.0)	26.8 (22.7) (0.0–100.0)	19.9 (17.6) (0.0–63.6)
	Question	18.7 (18.7) (0.0–100.0)	18.9 (17.4) (0.0–100.0)	18.3 (21.4) (0.0–100.0)	16.5 (16.2) (0.0–70.0)	19.2 (18.8) (0.0–100.0)	20.5 (21.4) (0.0–100.0)
	Positive appraisal	10.5 (13.3) (0.0–66.7)	9.4 (11.3) (0.0–50.0)	13.0 (16.5) (0.0–66.7)	10.6 (11.6) (0.0–50.0)	12.0 (14.5) (0.0–66.7)	7.5 (12.0) (0.0–59.0)
	Negative appraisal	8.6 (13.1) (0.0–100.0)	9.6 (13.7) (0.0–100.0)	6.5 (11.7) (0.0–66.7)	10.8 (11.6) (0.0–61.1)	9.5 (16.0) (0.0–100.0)	4.5 (8.1) (0.0–50.0)
	Pet-directed speech register (PDSR)	27.1 (21.8) (0.0–100.0)	28.6 (21.4) (0.0–94.7)	24.0 (22.6) (0.0–100.0)	23.8 (18.3) (0.0–65.1)	30.4 (24.6) (0.0–94.7)	25.5 (20.2) (0.0–100.0)
	PDSR - Pitch change	21.4 (19.6) (0.0–100.0)	24.0 (19.6) (0.0–94.7)	15.8 (18.7) (0.0–100.0)	20.3 (16.5) (0.0–64.0)	24.8 (22.0) (0.0–94.7)	16.8 (18.0) (0.0–100.0)
	PDSR - Slower tempo	9.6 (12.4) (0.0–75.0)	9.2 (11.8) (0.0–75.0)	10.3 (13.6) (0.0–75.0)	7.0 (8.6) (0.0–34.4)	9.1 (12.5) (0.0–75.0)	13.0 (14.8) (0.0–75.0)
	PDSR - Vowel modulation	17.6 (17.8) (0.0–100.0)	17.5 (16.4) (0.0–69.2)	17.8 (20.6) (0.0–100.0)	13.3 (12.1) (0.0–55.0)	20.8 (19.6) (0.0–83.3)	17.1 (19.3) (0.0–100.0)
	Irritation/frustration	0.9 (3.3) (0.0–30.0)	0.9 (3.6) (0.0–30.0)	0.7 (2.7) (0.0–16.7)	1.1 (3.7) (0.0–22.2)	0.8 (3.4) (0.0–30.0)	0.7 (2.7) (0.0–18.2)
	Mind reading	8.6 (10.6) (0.0–50.0)	9.0 (10.6) (0.0–50.0)	7.9 (10.7) (0.0–50.0)	6.7 (8.7) (0.0–35.7)	9.9 (11.7) (0.0–50.0)	8.9 (10.8) (0.0–50.0)
	Pet nickname use	13.4 (17.2) (0.0–100.0)	12.8 (15.4) (0.0–76.5)	14.7 (20.6) (0.0–100.0)	11.6 (15.2) (0.0–75.0)	13.0 (15.8) (0.0–100.0)	15.6 (20.8) (0.0–100.0)
	Parental role	2.3 (7.0) (0.0–66.7)	2.3 (7.2) (0.0–66.7)	2.4 (6.6) (0.0–33.3)	1.6 (4.2) (0.0–20.0)	2.9 (8.7) (0.0–66.7)	2.2 (6.6) (0.0–40.0)

Values in table are percentages (numerator = averaged frequency of variable of interest for a given participant, denominator = averaged frequency of “with pet” for a given participant). Values are presented as Mean (SD) (Line 1), and Range (Line 2).

### 4.6 Content reported in qualitative variables

Three variables in AURAL-Pet are qualitative (“other activity”, “vocalization type”, and “about what?”). Below are high-level descriptions of the content contained in each [for the full break-down of what each coder reported across all clips, see **Supplementary Data 1** (Raw Data)].

#### 4.6.1 Other activity

Coders were instructed to summarize pet-focused activities they deduced fell outside the scope of “playing” and “going on walk” (which were found to be two of the most common pet-focused activities in our sample, and the activities which we anticipated would be most indicative of the human-animal bond). The most frequent type of “other activity” reported by the coders concerned activities having to do with feeding or giving water to the pet. Phrases indicating this genre of activity included “feeding”, “giving treat” or “filling water”. The second most common “other activity” reported by the coders was training-related activities. Additionally, they described activities like going to the dog park, grooming (e.g., “giving bath”), and elimination (e.g., “pet bathroom break”, “going potty”).

#### 4.6.2 Vocalization type

Whenever pet vocalizations occurred, coders also reported the type of vocalization. The most common vocalization reported by the coders was “bark”, followed by “meow”, then “whine”, and “growl”. Cat purring was also reported frequently, as well as dog howling. Combinations of multiple vocalization types were also reported many times (e.g., “growl, bark”, “bark/whine”, “meow, bark”).

#### 4.6.3 About what?

Coders were instructed to summarize any “human substitute” content in a few words for each clip in which that variable was marked. The content in this variable varied the most (both between and within coders) out of the three qualitative variables. Most commonly, coders reported that the participants were either narrating to the pet their own actions or thoughts, or the actions and behaviors of the pets themselves (e.g., “pet’s actions”, “narrating actions”, “owner’s intentions”, “narrating”, “narrating thoughts”, “pet’s behavior”). Coders also reported that the participants spoke to pets about the weather multiple times (“temperature”, “weather”, “desert weather”), in addition to the “store”. They also reported that participants spoke to pets about future plans many times (e.g., “plans”, “doctor plans”, “future plans”). For further information about topics discussed by participants as reported by coders, see **Supplementary Data 1** (Raw Data).

## 5 Discussion

The AURAL-Pet coding system was created to quantify a variety of daily interactions between people and their cats and dogs as captured by the EAR. To do so, we created a coding system informed by theory and existing data, iteratively refined it via discussions amongst subject matter experts and feedback from real-time coders, and ultimately found it to be reliable between coders. We then retrospectively applied it to four real-world datasets.

As stated in the “Methods” section, all four of the studies that contributed the naturalistic datasets used in the current study were approved by the University of Arizona Institutional Review Board (IRB# 1803376749). Researchers who wish to apply AURAL-Pet to newly obtained audio data containing naturalistic interactions between people and pets should ensure adherence to local legislation and institutional requirements to guarantee participant privacy and informed consent.

We estimated inter-rater reliability to evaluate the degree to which AURAL-Pet’s variables were objective and feasible to implement on a diverse set of participants, as well as to ensure that our training protocol was sufficient. When estimating the reliability of the average codings (ICC[1,2]), we found that 22 out of 23 original AURAL-Pet variables met the EAR-standard threshold for acceptable agreement (ICC ≥.70). This demonstrates that almost all AURAL-Pet variables can be captured reliably using double coding (i.e., by averaging the coding from two coders). The one variable that did not meet the acceptable agreement threshold, “animal content”, should be interpreted cautiously when based on double codings. If this variable is central to addressing a core research question in future studies, investing in triple coding may be warranted. Furthermore, estimating the reliability of a single (ICC [1,1]) coding revealed that 14 out of 23 original AURAL-Pet variables are able to be reliably captured by just a single coder. Several high-level variables, such as the “activity” variables, “talking to pet”, “positive appraisal”, and “negative appraisal”, were all among those deemed to be reliably captured by a single coder (see **Table 2** for a full list of specific variables meeting this threshold). Thus, if resources for recruiting multiple coders are limited, future studies can be confident that focusing on these variables with a single coder still effectively and efficiently captures the variation in human-animal interactions between individuals. Studies which can dedicate resources to double coding will be able to reliably capture further nuance in these interactions, such as speaking to a pet as if they are human instead of an animal, if deemed important to answering the research questions (e.g., “do individuals who speak to their pets as if they are another human have stronger bonds with their pets than those who use more animal-level speech?”). Together, these findings inform future applications of AURAL-Pet by identifying which variables require additional coders to ensure reliable measurement.

Variables with the highest agreement (e.g., “pet vocalization”, “going for a walk”, “talking to pet”, and “question”; **Table 2**) were relatively simple for our coders to capture from the content of what was being said in the clips and/or did not allow for much subjective interpretation or ambiguity based on the ambient noises they were based upon. These variables tended to have relatively simple definitions, as well. On the other hand, variables with the lowest agreement (either by single or double coding) were generally those whose definitions contained unavoidable subjectivity. For example, each sentence or phrase that a participant communicated had to be marked as either “animal content” or “human substitute”. But what a coder considers “complex ideas that a pet could not comprehend” (see: “human substitute” in **Table 1**) naturally varies based on the coder’s judgment, and potentially even based on their own experience interacting with pets. Furthermore, while we can list some common examples, it is not feasible to list all possible topics a participant might talk about within each category. Even within this sample, there was plenty of variation in what was said to pets (see: “Content reported in qualitative variables” section). That being said, through our iterative process of testing and adjusting “animal content” and “human substitute” based on feedback from our coders, we reached a consensus and established that these variables are reasonably able to be captured. Additionally, as described above, all these limitations can be effectively addressed by double coding (or in the case of “animal content”, triple coding) the impacted variables. We therefore determined that they should be retained in AURAL-Pet as they have the potential to measure important nuance in the depth of human-animal interactions. We hope future research will validate whether differences in these interaction styles map onto different wellbeing outcomes and bonds with pets, as verifying those hypotheses was beyond the scope of the current study.

Another demonstration of a variable subjectivity limitation comes from our four pet-directed speech register variables (“pet-directed speech register”, “pitch change”, “slower tempo”, and “vowel modulation”). We struggled to define an objective cut-off for each of the three aspects of the speech register we included in AURAL-Pet. Future studies could consider using speech visualizing programs (e.g., PRAAT; 80), as past studies analyzing the pet-directed speech register have done (25, 30, 35, 38, 62). However, when coding a large number of human-pet interactions, capturing the essence of the speech register (i.e., either pitch change, slowed tempo, modulation to the vowel space, or some combination of the three) might be sufficient to capture this vocal register, particularly given the limited agreement on its defining features in the literature. This is reflected in the high agreement between our coders in the parent variable, “pet-directed speech register”. Additionally, future studies which are not limited to data in which some participants have as few as *n* = 2 clips in which pets are present, coders may be able to get a better sense of a participant’s baseline speaking register, and can therefore make more accurate judgments of what falls into the pet-directed speech register and what does not.

Along with limitations to AURAL-Pet introduced via subjective variable definitions, there are also some inherent limitations to auditory coding of short clips of human-pet interactions. For example, it is possible that coding only 30- or 50-second clips could lead to some interactions being cut off prematurely (81). However, this concern is partly addressed by the fact that we include multiple clips over time for each participant, and there is a robust literature that small slices of behavior can be quite predictive (82). Additionally, when coding auditory data with no visual component, ambiguity is introduced. Although our coders reached acceptable agreement (**Table 2**) for the variable “with pet”, the fact that the variable did not have perfect agreement means that even identifying whether a pet is present in each clip was not a given. We also initially attempted to identify clips which contained dogs or cats, but found that the only clips which coders could code with certainty were those in which specific pet vocalizations, like barking or meowing, occurred, and otherwise the two species were too difficult to distinguish reliably. We therefore dropped species from the final coding system. Furthermore, while we were generally able to identify whether participants were in proximity to a pet, we technically did not know if it was a pet they owned (a limitation of the archival nature of the data). Out of an abundance of caution, we therefore refer to participants as “pet-adjacent”, rather than “pet owners”, in this study. Fortunately, future studies that collect data on the pet-ownership status of their participants can easily address this limitation, including whether participants own cats or dogs specifically.

It should also be noted that because AURAL-Pet was created in the context of English-language interactions between people and dogs and cats, the coding system will need to be further assessed for reliability across different contexts (e.g., for interactions in other languages or with other pet species). Additionally, because all the audio data we analyzed was derived from the EAR, precautions should be taken when extending reliability estimates to audio data derived from other methods.

Despite some of these limitations, we nonetheless found that, according to thresholds previously set by EAR studies, all but one of the variables in AURAL-Pet can be reliably captured when averaged between two individuals (and over half can be reliably captured by one individual), and conclude that the quality of the coding system and the training methods outlined in the current manuscript are satisfactory for use in future studies. Additionally, researchers may use the fillable template and guide found in **Supplementary Table 4** (AURAL-Pet Template and Guide) to assist in data collection using AURAL-Pet.

Excitingly, applying AURAL-Pet to archival EAR datasets allowed us to determine a “base” rate of interactions with cats and dogs among different pet-adjacent populations. Crucially, since all pet-related analyses were done retrospectively on archival data that were not originally collected with the goal of analyzing human-pet interactions, there was no risk of social desirability effects pertaining to pet ownership. On average, participants spoke to dogs or cats in 9.2% of all clips in which they were speaking at all, demonstrating the non-trivial role that pets play in the lives of people. Furthermore, in applying AURAL-Pet to auditory data of human-pet interactions, we found that participants varied considerably in the quantity, quality, and depth of the interactions they had with the pets around them. For example, across 11 of our speech-based interaction variables, some participants demonstrated these behaviors in none of their clips, while other participants demonstrated them in every clip (**Table 4**). Although the exploratory nature of the current study and our lack of a-priori hypotheses preclude us from making strong claims regarding differences in how various demographic groups interact with pets, the varied results between different age brackets and genders lay the groundwork for analyzing differences in how people interact with their pets across different contexts. The variability in interactions both between and within demographic groups could explain the heterogeneity in the impact of pet ownership on mental health and wellbeing across human-animal interaction studies (5, 12). In the future, this hypothesis can be assessed by mapping differences in interaction styles between individual participants onto independent measures of mental health, wellbeing, and the human-animal bond.

We are hopeful that future studies will utilize AURAL-Pet to quantify variability in human-pet interactions, and that this variability may further elucidate the role of pets in human mental health and wellbeing. The implementation of AURAL-Pet could be especially applicable for facilitating understanding of the role pets play in promoting positive human mental health and wellbeing. For example, if studies find that individuals who use more “positive appraisal” with their pets have better mental health outcomes, this could provide evidence for the social support hypothesis in which pet ownership may improve pet owners’ mental health through providing positive social interactions. Similarly, the variable “talking about pet” could potentially be used as a proxy to understand how pets act as a social catalyst to promote social interaction with others (4). As another example, by quantifying both instances of “positive appraisal” and “negative appraisal”, we can investigate whether having a pet that displays more perceived “negative” behaviors correlates with worse human mental health outcomes (e.g., social norms hypothesis; 4).

AURAL-Pet can also be used as a complementary tool alongside other methods of capturing behavior to explore how audio-based human-pet interactions correlate with other forms of social and behavioral engagement. For example, AURAL-Pet could be combined with a visually-based human-animal interaction coding system (e.g., the Observation of Human-Animal Interaction for Research [OHAIRE] coding system, which measures behaviors like touching, gesturing, and smiling; 83) to represent a more holistic picture of human-animal interaction accounting for both vocal and visual behaviors. Additionally, since AURAL-Pet captures both positive and negative human-pet interactions with limited bias, this tool may begin to help address measurement limitations in the field of human-animal interaction in which scales of the human-animal bond tend to lack standardization and reliability (84, 85). By quantifying the valence and depth of interactions, AURAL-Pet can be explored for use as a method of validating subjective owner responses on surveys concerning the human-animal bond, which tend to skew positively. Furthermore, since many people view their pets as integrated members of their family (including as a proxy for their children; 86), measuring if these individuals also demonstrate higher rates of “human substitute” and “parental role” may provide a more objective measure of this phenomenon. Finally, in future EAR studies, coders can apply AURAL-Pet alongside other EAR coding systems, such as the Social Environment Coding of Sound Inventory (SECSI; 87), to better understand how the quantity and depth of human-pet interactions associate with the quality and depth of human social interactions. Together, these methods could assist in answering questions like whether individuals who speak less frequently with other humans make up for this lack of interaction by speaking more often with their pets (88).

In summary, AURAL-Pet is a novel coding tool which can be used to quantify auditory human-pet interactions. We found the system was reliable across a diverse range of ages, genders, and interaction types. Importantly, AURAL-Pet fills a crucial gap by capturing the nuances of the quality and depth of spoken content towards pets. Such interactions are pervasive in our society, and understanding them may have wide-ranging implications for psychiatry, psychology, sociology, and gerontology, as well as human-animal interaction research, animal behavior, and veterinary medicine. In future studies, AURAL-Pet can be used in conjunction with mental health and wellbeing measures to explore how individual differences in the ways in which people interact with their pets may moderate the relationship between pet-ownership and wellbeing at both ends of the human-pet dyad.

## Data Availability

The original contributions presented in the study are included in the article/**Supplementary Material**. Further inquiries can be directed to the corresponding author.
