# Supplementary material for: Cross-species conversations: the development of the AURAL-Pet coding system to capture how people talk to pets in daily life
Source: Front Psychiatry. 2026 Aug 4;17:1881870. doi: 10.3389/fpsyt.2026.1881870 (PMC13482966; doi:10.3389/fpsyt.2026.1881870)
Supplement: Supplementary file 1 [file Table3.docx]

**Supplementary Table 3.** Pet-adjacent participant demographics by study.

|  | **Study A –**  **Aging**  (N = 49) | **Study B –**  **CALM**  (N = 81) | **Study C –**  **Cancer**  (N = 28) | **Study D –**  **DSE**  (N = 82) | **Overall**  (N = 240) |
| --- | --- | --- | --- | --- | --- |
| **Age** |  |  |  |  |  |
| Mean (SD) | 75.4 (6.1) | 33.0 (8.5) | 54.2 (12.6) | 45.9 (10.0) | 48.5 (17.9) |
| Median  [Min, Max] | 76  [65, 87] | 30  [25, 55] | 55  [24, 81] | 47  [25, 65] | 47  [24, 87] |
| **Gender** |  |  |  |  |  |
| Female | 27 (55.1%) | 57 (70.4%) | 19 (67.9%) | 61 (74.4%) | 164 (68.3%) |
| Male | 22 (44.9%) | 24 (29.6%) | 9 (32.1%) | 21 (25.6%) | 76 (31.7%) |
